# The United States–Mexico border environmental public health: the challenges of working with two systems

**DOI:** 10.26633/RPSP.2017.98

**Published:** 2017-07-20

**Authors:** Genny Carrillo, Felipe Uribe, Rose Lucio, Alberto Ramirez Lopez, Marcelo Korc

**Affiliations:** 1 Department of Environmental and Occupational Health School of Rural Public Health McAllen, Texas United States of America Department of Environmental and Occupational Health, School of Rural Public Health, Texas A&M Health Science Center, McAllen, Texas, United States of America.; 2 El Colegio de la Frontera Norte, Coahuila El Colegio de la Frontera Norte, Coahuila Coahuila Mexico El Colegio de la Frontera Norte, Coahuila, Mexico.; 3 Border Environmental Cooperation Commission Border Environmental Cooperation Commission El Paso, Texas United States of America Border Environmental Cooperation Commission, El Paso, Texas, United States of America.; 4 Sustainable Development and Human Security Pan American Health Organization Washington, D.C. United States of America Sustainable Development and Human Security, Pan American Health Organization, Washington, D.C., United States of America.

**Keywords:** Border areas, border health, bilateral cooperation programs, environmental health, delivery of health care, Mexico, United States, Áreas fronterizas, salud fronteriza, programas de cooperación bilateral, salud ambiental, prestación de atención de salud, México, Estados Unidos, Áreas de fronteira, saúde na fronteira, programas de cooperação bilateral, saúde ambiental, assistência à saúde, México, Estados Unidos

## Abstract

This report shares the challenges and opportunities encountered by a binational project that examined the availability of environmental and public health information for the United States–Mexico border area. The researchers interviewed numerous national and binational agencies on both sides of the border, endeavoring to develop a framework to advance the knowledge of academic and public health professionals in the area of environmental border health. However, the lack of standardized indicators and metrics in both countries validates the emergent need to establish a viable framework for the collection, analysis, and dissemination of environmental information. Recommendations for next steps are included.

## RESUMEN

En este informe se describen los retos y las oportunidades que ha detectado un proyecto binacional que examinó la disponibilidad de información sobre salud ambi-ental y salud pública en la zona fronteriza entre México y los Estados Unidos. Los investigadores entrevistaron a numerosos organismos nacionales y binacionales a ambos lados de la frontera, a fin de elaborar un marco para la difusión del conoci-miento de los profesionales académicos y de la salud pública en el ámbito de la salud ambiental fronteriza. La falta de indicadores y métodos de medición normalizados en ambos países confirma la necesidad emergente de establecer un marco viable para la recopilación, el análisis y la difusión de información ambiental. Se incluyen recomen-daciones para los próximos pasos.

## RESUMO

Este relatório apresenta os desafios e oportunidades encontrados por um projeto binacional que examinou a disponibilidade de informações ambientais e de saúde pública na região da fronteira entre o México e os Estados Unidos. Os pesquisadores entrevistaram diversas agências nacionais e binacionais em ambos os lados da fronteira, procurando desenvolver um sistema destinado a promover os conhecimentos de profissionais acadêmicos e de saúde pública na área da saúde ambiental em regiões de fronteira. No entanto, a falta de indicadores e métodos de medição padronizados entre os dois países destaca a necessidade emergente de estabelecer um sistema viável para a coleta, análise e difusão de informações ambientais. São feitas recomendações sobre as próximas medidas a serem tomadas.

The United States–Mexico border area spans 3 100 km. In 2010, its population was more than 14 608 655 million people, with 7 303 754 million on the United States side and 7 304 901 million on Mexico side (1). At current growth rates, the population is set to double in approximately 35 years, reaching an estimated 29 million residents by 2045 (2).

**FIGURE 1.**
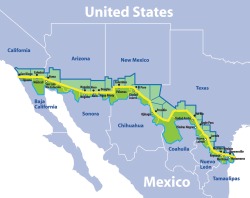
United States–Mexico border area showing its “sister cities,” 2016.

***Source:*** Pan American Health Organization. Transnational cooperation in environmental health along the US-Mexico border. Washington, DC: PAHO; 2013. Reproduced with permission.

The border area has 14 pairs of “sister cities” that are linked to one another both economically and culturally, and in many cases, share waterways (Figure 1). These sister cities have many issues in common, but also differ substantially in how they are governed; their access to health care; and their priorities for disease surveillance and environmental data collection (3–9).

The United States and Mexico are facing critical gaps in environmental health knowledge that could compromise binational efforts to confront diseases related to environmental pollutants. Many of the environmental health hazards faced by the United States–Mexico border states are unknown due to a lack of basic data that would allow comparisons among the environment and health indicators (6). Due to the health disparities among border populations, it is imperative that information sets and databases from both countries be identified and made accessible to the public health professionals in the area (10, 11).

Preventing and reducing chronic and infectious health problems—especially asthma, birth defects, cardiovascular disease, diabetes, and pediatric cancers that strike hundreds of thousands of families in both countries annually—demands shared information. Currently, to advance binational knowledge, academics and public health professionals on both sides work independently or collaboratively on various environmental or health topics, but must gather available information from numerous different sources, ranging from peer-reviewed journal articles to local agencies in both countries (12).

Additionally, the United States–Mexico border area is plagued by international and domestic policy issues involving lawmakers and law enforcement officials in both countries (13). The number of people who cross the border every day potentially increases the risk of transmitting communicable diseases from one country to the other. As such, several binational agencies and programs have been created with different missions and goals to address the challenges and facilitate collaborative work among the United States and Mexico in the border area (14).

The purpose of this project was to identify the most important and prevalent environmental health issues in the United States–Mexico border area and to identify a stakeholder task force. This report, however, focuses on the many challenges and opportunities encountered by the study it sought to identify border agencies that generate and share health and environmental data for analyses. Ultimately, the goal was to develop a framework for a multi-phase process that would produce a binational environmental and health tracking model to be replicated throughout the border area.

## MATERIALS AND METHODS

This project was conducted in 2014 – 2015 and focused on Hidalgo, Kinney, and Maverick counties in the State of Texas in the United States, and Piedras Negras municipality in the State of Coahuila in Mexico. Thirty local and international stakeholders from both countries were contacted and surveyed (Table 1) using an open-ended interview that covered methodologies for data collection, management, and accessibility, and type of data on environmental contaminants, as well as impact on population health. A content analysis of interviews was performed.

To establish partnerships with binational stakeholders, the researchers first drew up “Memorandums of Understanding” with authorities from Mexico and the United States to ascertain the data type and collection methodology being employed by each. The Mexican agencies approached were the *Secretaría de Medio Ambiente y Recursos Naturales* (Environment and Natural Resources Secretariat; SEMARNAT), the *Secretaría de Salud* (Health Secretariat), as well as other state and federal agencies. The United States agencies were the Department of Health and Human Services (DHHS), the Environmental Protection Agency (EPA), the Texas Commission on Environmental Quality (TCEQ), and other potential sources, including the 11 public health regions that compose the Texas Department of State Health Services. A number of key partners were identified from among various local and regional governmental agencies and from academic institutions in the State of Tamaulipas (Mexico) and Texas. Table 2 lists some of the contributors based on their access to data for this project.

### Source information in Mexico

An integrated system will assist various governmental and non-governmental organizations in Mexico. Data collected in Mexico regarding health and environmental issues is managed by federal and state authorities. The Health Secretariat organizes and operates the *Sistema Nacional de Vigilancia Epidemiológica* (National System for Epidemiological Surveillance; SINAVE), which identifies disease cases, mortality, and risk and protective factors for epidemiological surveillance according to national standards (15).

It is important to clarify that epidemiological surveillance in Mexico is integrated in the *Sistema Único de Información para la Vigilancia Epidemiológica* (Information System for Epidemiological Surveillance; SUIVE). This system has four components: Weekly New Cases (SUAVE); the Hospital Network for Epidemiological Surveillance (RHOVE); the Epidemiologic and Statistic Systems for mortality; and the Epidemiologic Surveillance for Special Systems. It is important to mention that operations and procedures for epidemiologic surveillance are standardized through out the different institutions of the National Health System (16).

SUAVE is a weekly reporting system of new cases of diseases in Mexico. The morbidity data gathered can be analyzed at various levels—local, state, regional, and by jurisdiction. For this report, the data was gathered and analyzed at the “Sanitary Jurisdiction Level I” for the City of Piedras Negras, Coahuila. This jurisdiction comprises several municipalities located in the northern part of the State of Coahuila, including Allende, Guerrero, Hidalgo, Nava, Piedras Negras, and Villa Union. In 2010, these municipalities had 202 888 inhabitants (17). In Mexico, the methodology for calculating an indicator is based on the total number of cases for a specific disease divided by the total population covered by the health system. Thus, the crude incidence age-adjustedrate is obtained using the straightforward method of adjusting rates (18).

**TABLE 1.** Description of national and binational agencies charged with health and/or environmental oversight in the United States of America and Mexico, 2016

Agency	Description of activities	Website
UNITED STATES OF AMERICA	
Environmental Protection Agency	A federal agency created to protect human health and the environment by developing and enforcing regulations based on laws passed by the United States Congress. It provides environmental assessments, research, and education, and maintains and enforces national standards under a variety of environmental laws, in consultation with state, tribal, and local governments.	http://www3.epa.gov
Department of Health and Human Services	Collects national health information through the Centers for Disease Control and Prevention and the National Center for Health Statistics.	https://www.hhs.gov
Texas Department of State Health Services	Comprised of seven offices that focus on the state's mental health, substance abuse, finances, operations, family and community health services, regulatory services, regional and local services, and disease control and prevention. Reporting of infectious disease cases is mandatory.	http://www.dshs.texas.gov
Texas Commission on Environmental Quality	Strives to protect the state's public health and natural resources in conjunction with sustainable economic development and to provide clean air and water and safe waste management.	https://www.tceq.texas.gov
Centers for Disease Control and Prevention (CDC)	Collects and uses public health surveillance data to protect the country's health and safety by controlling and preventing disease.	https://www.cdc.gov/
National Notifiable Disease Surveillance System	Staff works closely with state and local health departments; experts from other CDC programs; and partners	https://wwwn.cdc.gov/nndss/
Council of State and Territorial Epidemiologists	Provides technical advice and assistance to partner organizations and to federal public health agencies	http://www.cste.org/
BINATIONAL	
The Border Environment Cooperation Commission	Created in 1994 by the federal governments of the United States and Mexico as a side agreement to the North American Free Trade Agreement. Along with its sister-institution (the North American Development Bank), it strives to improve the environmental conditions of the Mexico-United States border area for the well-being of residents in both nations.	http://www.becc.org/
The United States-Mexico Border Health Commission	Created in July 2000 by the United States Secretary of Health and Human Services and the Secretary of Health of Mexico and designated as a Public International Organization by the United States in 2004. It has the unique opportunity to bring together the two countries and its border states to solve border health problems.	http://www.borderhealth.org/
MEXICO
Secretaría de Medio Ambiente y Recursos Naturales	A federal agency that strives toward protection, restoration, and conservation of natural resources, most importantly ecosystem conservation and biodiversity, pollution prevention and control, water resource management, and climate change mitigation.	http://www.gob.mx/semarnat
Secretaría de Salud	A federal agency that integrates and processes information from various institutions that belong to the National Health System, which provides national statistics. Organizes and operates the Sistema Nacional de Vigilancia Epidemiológica, which defines disease cases, mortality, and risk and protective factors that constitute the nation's standards for epidemiological surveillance.
Secretaría del Medio Ambiente del Estado de Coahuila	Emissions inventory for the evaluation of air quality. Registry of emissions and pollutants transfer.	http://www.sema.gob.mx/SGA-MONITOREO-EMISIONES2008.htm

**TABLE 2.** List of Mexican stakeholders interviewed and scope of work related to environmental and health issues in the northern part of Coahuila State, Mexico, 2016

Stakeholder	Administrative level
Federal Office of Environmental Protection	Municipal and State
Ministry of the Environment of Coahuila	Municipal and State
Ministry of the Environment and Natural Resources	Regional, State, and Federal
Directorate General of Municipal Ecology	Municipal
Center for Rural Development Support	Regional
Municipal Health Office	Municipal
Municipal Ecological Regiduría	Municipal
Ecological Group "Friends of Río San Rodrigo"	Municipal
Ecological Group "Green Tec Osos"	Municipal
Sanitary Jurisdiction I (regional health authority)	Regional
Municipal Water and Sanitation System	Municipal
Meteorological Observatory (National Water Commission)	Municipal
Office of Sanitary Inspection (Customs)	Federal
International Waters Commission	Federal
Institutions of the National Health System	Federal and State
Autonomous University of Coahuila	State

RHOVE gathers information about hospitalization discharges, but only includes hospitals that are part of the Health Secretariat. The information obtained is analyzed by the hospital. In the northern part of the State of Coahuila, data on hospital discharges is gathered from the General Hospital in Piedras Negras. In order to calculate indicators, the total number of hospital discharges are registered by year per disease, divided by the total discharges in the year multiplied by 100.

Considering the importance of timely and accurate information on health conditions and the need to understand the prevalence and distribution of target conditions for both sides of the border, the researchers examined relevant existing and potential health data sources at the national, state, and regional levels. Additionally, current national and state health tracking systems were reviewed to explore the potential opportunities and constraints of their data sources in providing integrated information for effective assessment and analysis of community health.

Identification of common indicators in the United States–Mexico border area is urgently needed; however, it will take time to develop policies and procedures for addressing the issue. Health and environmental health professionals and federal representatives from both countries participated in a forum held on 6 February 2014 in McAllen, Texas, for the purpose of gathering input from participants on their respective and most significant environmental and health issues. Topics such as water quality and gastrointestinal diseases and air pollution from unpaved streets were acknowledged. The participants also provided information on the agencies related to each topic; however, they were unable to speak to the quality of the data required nor how to gather it.

## RESULTS

The results of this project show that morbidity indicators from both sides of the United States–Mexico border area cannot be compared because the metrics used in data collection differ greatly. For example, in Coahuila, morbidity from asthma should be an indicator registered in the National Health System, but it is not. In Texas, there are annual surveys that evaluate diseases such as asthma and the data can be stratified using different population characteristics. In Coahuila, the indicator of annual hospital discharges by a specific disease is calculated based on the total number of hospital discharges in a given year. In Texas, there is a registry for annual discharges in a county and the indicator is comprised of a numerator of total health cases and a denominator of the exposed population.

In terms of the environment, the State of Texas utilizes air monitoring stations that are located near the United States– Mexico border. These stations are run by TCEQ and measure ranges of PPM 10 and PPM 2.5. In Coahuila, there were two data sets of air pollution taken in 2005 and 2008. These data were crude, measured in tons of air pollutants freed into the air and accumulated per year. The lack of standardized indicators and metrics in both countries validates the emergent need to establish a viable framework for the collection, analysis, and dissemination of environmental information. Currently, some programs, such as the United States–Mexico Border Health Commission are producing United States–Mexico data reports, but provide information/data sets separately (one for the United States, another for Mexico) due to the differences in data collection measures and metrics, and lack of any standardization.

Although Mexico and the United States share several common interests related to environmental and health problems along the border, there are conflicting positions on how to address them due to the absence of a legal framework on which to build bilateral cooperation. The creation of the United States–Mexico Border Health Commission (BHC) and the Border Environment Cooperation Commission (BECC) acknowledged the need for structures and mechanisms for fostering bilateral cooperation. A focus of these should be to develop a governance approach that includes coordinating among the various interests and stakeholders to assist in clarifying direction related to government’ actions (19).

When the Governments of Mexico and the United States created the BHC in 2000, one of the objectives was to “strengthen the system of border health information” (20). However, a health information system was not among its priorities or initiatives. Nonetheless, an administrative group that emerged at the local level was the Bi-national Health Council (BiHC). Originally sponsored by the Texas Department of State and Human Services, the BiHC extends this group along the international border (20). This regional group forms a local framework for acting on bi-national health issues, establishing several priorities in the field of public health. However, these bi-national health councils do not include research in their objectives, and as such are not gathering comparable information on health issues. Consequently, these bi-national health councils should serve as the administrative figures from which BHC could meet its objective of strengthening the border health information system. Additional support could be garnered from other actors, such as the Working Group on Border Health Research, which is part of the BHC, and the Group of Leaders Across Borders in Arizona and Texas, among others.

BECC meanwhile, supports initiatives to solve critical environmental and health conditions in the border area in spite of the lack of environmental infrastructure (6). Thus, BECC is another administrative figure from which regional actors could develop projects in order to create infrastructure and utilize equipment to obtain comparable information from a bi-national perspective.

## DISCUSSION

The framework of any study needs to consider that data collection and health and environmental resources exist in a cultural, organizational, and social context that should be included in the analysis (21). In particular, this complexity exists in a cross-border area where political, administrative, economic, and cultural processes converge and are expressed differently by each country. It is therefore, imperative to analyze the decision-making processes of each country that could directly or indirectly affect the development of information on health and the environment and its accessibility. It is necessary to consider the interaction of numerous factors and to address how information should be handled and shared.

Tremendous progress was made toward improving the quality of the environment and human health in the United States–Mexico border through the Border 2012 Program and is being continued through the 2020 Program (22). Academics and public health professionals are working together to improve border health and develop some type of surveillance for a range of important topics, including infectious diseases (23, 24). However, much more collaboration, access to data, and a similar report system are needed to link environmental factors to environment-related health issues. Implementation of a bi-national environmental health tracking system along the border is important as it will help identify environmental health exposures among the communities, as well as related health consequences.

### Challenges

It became apparent during the course of the project that “Memorandums of Understanding” were not sufficient for obtaining immediate access to the requested information. Moreover, when obtained, the information from different institutions was incomplete, partial, and limited in relation to geographic distribution and time periods. This is contrary to the norms that regulate the quantity and quality of the gathered information.

Furthermore, the process of interviewing institutional agencies and nongovernmental organizations that collect and manage health and environmental information made it evident that staff charged with data often did not have decision-making authority. Although an executive at the contacted institution might have agreed to provide the required information, the task was often delegated to a subordinate and the outcome differed from the expectation set.

On the other hand, the information garnered from interviews of health and environmental authorities shed light on the most important health consequences related to pollutants. However, they were uncertain on how to access the data that supported their statements or whom to contact to obtain it. In addition, they pointed to a lack of standards among sources, contents, the extent of, and quantity of the information mentioned.

It is also important to mention that the United States faces challenges related to data collection at the state and county levels since much of it comes from investigations of outbreaks that are resolved locally. All too often this data is not reported to the appropriate entities, and thus, is not included in national databases.

Government agencies from both countries are charged with addressing the environmental and health challenges arising from the growing population and the industrialization of the United States–Mexico border area. A clearer path is needed within the agencies to help academics and researchers identify where and how to gather information required to study the border area’s specific and diverse public health problems.

### Recommendations

Based upon the 2-year experience of this study, it is extremely important that researchers who wish to engage in a similar study first develop a formal plan or strategy for working with binational agencies to: (a) identify entities/individuals with whom to work; (b) gather information; and (c) interpret and compare the collected data. Researchers from the United States are more readily available, and fortunately, data is more easily obtained from many websites, as well as through requests for information made to State and Federal agencies.

The Pan American Health Organization (PAHO) has developed a socioecological model that proposes a four-step public health approach to reduce and prevent transnational environmental health problems along the United States– Mexico border area (10). This model should be used as the basis for establishing a standardized binational data collection program based on the environmental health priorities of both countries.

The development and identification of common binational environmental health issues and collaboration is imperative for the success of any initiative. It is important to specify which agencies will lead the project, and if possible, which departments within the collaborating agencies will be responsible for the provision of information. It is also of paramount importance that authorized contacts be identified, due to restrictions limiting access to databases and information. Nonetheless, setbacks in an established network may still occur if government policy and/or priorities change.

### Conclusions

The United States–Mexico border populations shares many environmental, social, economic, cultural, and health characteristics. However, addressing their everyday problems is complicated by differing rules and regulations pertaining to the political and administrative organizations of their respective countries. This has resulted in health and environmental problems that are addressed unilaterally by each country’s institutions, undermining interagency cooperation for data collection and analysis and shared solutions. Future studies should focus not only on the governance of the information generated in each country, but also on how to resolve border problems through cooperation at the regional level.

Many border programs have been developed and work with public health professionals to address environmental and health issues; however, more is needed to collect, integrate, analyze, and disseminate standardized data from both countries. Furthermore, it is important that this data be contained within one main database that can be accessed by both countries. Additionally, standardized metrics would allow both countries to compare and contrast statistics in a more efficient and reliable manner. These metrics would also facilitate better policy development to provide improved public health for the border areas in the 21st Century. Building on the strengths of each country and taking into consideration the limitations of the current modes of cooperation will bring forth a new era characterized by partnerships among truly binational border-wide organizations and stronger collaboration between the public health and environmental protection sectors, which will lead to a better future for the United States–Mexico border.

Although it is unrealistic to believe that every single disease will be tracked and that all information will be shared bi-nationally, if health officials from both countries identify and categorize priority diseases and environmental issues, a standardized data set could be developed and monitored. High ranking health and environmental officials need to work closely to identify venues to develop an environmental public health system that will help both countries institute policies to decrease environmental exposure and to monitor the possibility of outbreaks. It is important to develop binational or international mechanisms utilizing organizations, such as PAHO, to develop databases and tools to share information. Such a collaboration may provide governmental agencies with confidence regarding the storage, handling, exchange, and dissemination of information on health and the environment. The latter is becoming increasingly strategic from a political standpoint and will be more critical as the effects of climate change are experienced, especially in communities along the United States–Mexico border.

#### Acknowledgements.

The authors wish to acknowledge to Juan Parra, of El Colegio de la Frontera Norte for assisting with the editing of the bibliography.

#### Funding.

This project was carried out with funds obtained from the Border Environmental Cooperation Commission (Award Number: TAA14-026; B2020 R6.934).

#### Disclaimer.

Authors hold sole responsibility for the views expressed in the manuscript, which may not necessarily reflect the opinion or policy of the *RPSP/PAJPH* and/or PAHO.
